# Endometriosis—A Multifaceted Problem of a Modern Woman

**DOI:** 10.3390/ijerph18158177

**Published:** 2021-08-02

**Authors:** Alicja Mińko, Agnieszka Turoń-Skrzypińska, Aleksandra Rył, Patrycja Bargiel, Zuzanna Hilicka, Kaja Michalczyk, Paulina Łukowska, Iwona Rotter, Aneta Cymbaluk-Płoska

**Affiliations:** 1 Department of Medical Rehabilitation and Clinical Rehabilitation, Pomeranian Medical University, ul. Żołnierska 54b, 71-210 Szczecin, Poland; agi.skrzypinska@gmail.com (A.T.-S.); ryl.ola@gmail.com (A.R.); iwrot@wp.pl (I.R.); 2 Student Science Club “KINEZIS”, Department of Medical Rehabilitation and Clinical Physiotherapy, Pomeranian Medical University, 71-210 Szczecin, Poland; bargiel2727@o2.pl (P.B.); hilickaz@gmail.com (Z.H.); 3 Department of Gynecological Surgery and Gynecological Oncology of Adults and Adolescents, Pomeranian Medical University, al. Powstańców Wielkopolskich 72, 70-111 Szczecin, Poland; kajamichalczyk95@gmail.com (K.M.); lukowska.paulina95@gmail.com (P.Ł.); anetac@data.pl (A.C.-P.)

**Keywords:** endometriosis, physical activity, depression, anxiety, sexual function, coping with stress

## Abstract

Endometriosis is a chronic disease of unclear aetiology that affects millions of women around the world. It causes chronic pain, dysmenorrhea, and infertility, which significantly reduces the quality of daily life. The aim of the following study was a multivariate analysis of the functioning of women diagnosed with endometriosis, and the identification of the relationship between the level of physical activity and sexual functioning, ability to cope with stress, and the degree of anxiety and mood disorders. The prospective survey was conducted of 957 women. The research was carried out using standardised IPAQ, FSFI, HADS, and Mini-Cope questionnaires. The study showed that patients with endometriosis exhibit a higher level of depression and anxiety disorders (*p* = 0.01) and a lower level of sexual functions (*p* < 0.001). The influence of physical activity on the functioning of patients with endometriosis was demonstrated. Depending on the clinical stage of endometriosis, the influence of physical activity on individual aspects of life differed. Physical activity was shown, inter alia, to reduce anxiety (*p* = 0.015), and influence stress-coping strategies. Endometriosis affects the mental and physical health of women. Physical activity can reduce the severity of endometriosis symptoms and improve the daily functioning of patients.

## 1. Introduction

Endometriosis is a chronic, progressive disease of unclear aetiology. It is characterised by the growth of endometrial tissue outside the uterine cavity and chronic inflammation of the affected anatomical structures—most often the pelvic organs. It is one of the most common gynaecological diseases [1,2]. It is estimated that the incidence of endometriosis ranges from 2 to 17% of women of reproductive age, which represents over 170 million women globally [3,4]. In Poland, the disease affects about 1 million people [5]. The rate of diagnosed cases is much higher among women with infertility (25–50%), dysmenorrhea (40–60%), and pelvic pain (71–87%) [6,7]. The pathomechanism of endometriosis is based on several theories. The main one is the theory of retrograde menstruation, which is the movement of endometrial tissues through the fallopian tubes during menstruation. Other theories of the development of endometriosis are related to genetic predisposition, environmental factors, immune disorders, and lifestyle [8,9].

With regard to recent literature, the non-modifiable risk factors for endometriosis include age, family history, education level, and the age of the first menstruation (menarche). No association between lifestyle factors, such as alcohol/caffeine consumption or smoking, and the incidence of endometriosis has been established. A weak correlation between the lack of physical activity and an increased risk of endometriosis has been demonstrated [10,11]. The most common symptoms of the disease include dysmenorrhea (60–80%), chronic pelvic pain (30–50%), infertility (30–40%), and dyspareunia (25–40%) [12]. In accordance with the classification introduced by the American Society for Reproductive Medicine (ASRM), there are four stages of endometriosis. The relationship between the staging of endometriosis and the pain severity remains unclear [13,14]. In accordance with the recent literature, there is evidence that the age of women with advanced-stage endometriosis was slightly higher at diagnosis. The highest incidence was recorded among women aged 25–29, whereas the lowest was among women aged over 44 [15,16]. Moreover, the symptoms were found to alleviate in postmenopausal women due to reduced oestrogen production [17]. Endometriosis is difficult to diagnose due to the gaps in knowledge among healthcare professionals about the condition. This applies especially to uncertainty regarding the pathogenesis, heterogeneity of the course of the disorder, or its symptoms, which are often confused with other mental or physical disorders. Due to the aforementioned factors, the diagnosis of endometriosis is often overlooked, which is a serious social problem [12,18,19]. Treatment based on pharmacological methods relieves the symptoms of the disease but may involve the occurrence of side effects and a recurrence of symptoms after discontinuation of use. Laparoscopy, the purpose of which is ablation or excision of lesions, is considered the gold standard in the diagnosis and treatment of endometriosis [9]. The pharmacological and surgical treatment significantly affects the quality of life of patients with endometriosis; however, there are multiple factors that can significantly affect the functioning of women. Women suffering from endometriosis can experience a variety of non-clinical symptoms. Endometriosis has been found to have a negative impact on physical, mental, and social well-being [20,21]. The pain associated with endometriosis is the key factor that reduces the quality of life because it can lead to reduced sleep quality, more perceived stress, lower levels of physical activity, and mental disorders, such as anxiety and depression. Moreover, it negatively influences sexual activity, which has further consequences for intimate and social relations [22,23]. The adverse effects experienced by patients with endometriosis significantly contribute to problems with productivity, relationship difficulties, and social dissatisfaction. Endometriosis is therefore a pathology that can significantly affect the quality of women’s life in multiple dimensions [1,24,25]. Modern treatment of endometriosis should be personalised using a patient-centred, interdisciplinary approach. Pharmacological treatment may not be sufficient. Complementary to the therapy, a psychological, dietary, urological, and physiotherapy intervention is recommended [26,27,28].

The aim of this study was to determine the impact of endometriosis on women’s functioning, and to identify the mutual relationship between the level of physical activity, sexual functioning, and the degree of anxiety and depression disorders among women with endometriosis. Moreover, the study also included the analysis of the relationship between the level of physical activity and sexual functioning, coping skills, and the degree of anxiety and mood disorders, taking into account the clinical advancement of endometriosis.

## 2. Materials and Methods

### 2.1. Survey Sample

The prospective study was conducted from March 2019 to April 2021 and included 957 women. The respondents were divided into two groups: study group (B) and control group (C). The group B consisted of 484 people, and the group C of 473. The minimum sample size was 384. The inclusion criteria for the study in both groups were the age from 18 to 55 years and fully completed questionnaires (FSFI, HADS, IPAQ, and Mini-Cope). Only women with clinically diagnosed endometriosis were included in group B. Group C was composed of women without endometriosis. It was used as a reference for the analysis of the results in group B. The literature data [29,30,31] shows that there are multiple factors, including the stress level, level of physical activity, depression and anxiety disorders, and sexual functioning, which differ in terms of groups of women with and without endometriosis.

**Figure 1:**
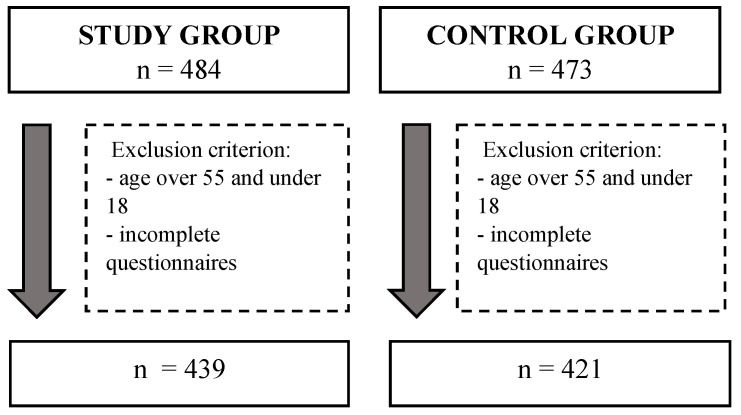
Flowchart.

Minors and persons over 55 were excluded from the study. Women with concomitant diseases, such as rheumatoid diseases, hypertension, atherosclerosis, diabetes, infertility, epilepsy, and gynaecological problems, treatment of which could have an impact on the assessment of functioning and quality of life, could not take part in the study. Persons who did not reply to all of the questions included in the questionnaires were also excluded. Only complete questionnaires were used in the analysis. All questionable data were discarded. Ultimately, taking into account all of the inclusion and exclusion criteria, 439 women qualified for group B, and 421 for group C (Figure 1). Group B was recruited through internet forums associating women with endometriosis. Group C was gathered via publicly available social networks. The study collected demographic data and data on risk factors and the course of the disease, such as: age, education, the occurrence of addictive behaviours (smoking and drinking alcohol), the course of the menstrual cycle, multiple pregnancies, and the occurrence of miscarriage. Women diagnosed with endometriosis were additionally assessed in terms of the location of the foci and the presence of accompanying symptoms. The above variables were treated only as information concerning the group characteristics and they were not taken into account in further analyses.

### 2.2. Questionnaires

The survey was conducted using standardised questionnaires: International Physical Activity Questionnaire (IPAQ), Female Sexual Function Index (FSFI), Mini-Cope, and Hospital Anxiety Depression Scale (HADS) translated into Polish. 

The level of physical activity was assessed using the IPAQ questionnaire, which expresses physical activity in MET-minutes/week units. On the basis of the obtained results, the study participants could be classified according to their level of physical activity into one of three categories: high (over 1500 or 3000 MET-minutes/week), sufficient (600–1500 or 600–3000), or insufficient (below 600 MET-minutes/week) [32,33]. The IPAQ questionnaire demonstrated acceptable accuracy and reliability in the assessment of physical activity among adults. The criteria validity had a rho median that approximately equalled 0.30, which was comparable with most other self-reported validation studies [34,35].

Female sexual function was assessed using the FSFI questionnaire, the purpose of which was to determine significant clinical sexual dysfunctions. The questionnaire consists of 19 questions, divided into 6 domains, assessing such factors as: desire, arousal, lubrication, orgasm, sexual satisfaction, and pain related to sexuality [18]. The overall FSFI score ranges from 0 to 36 points. The higher the score, the better the sexual function. Women with a score of 26 or less were classified as having sexual dysfunction [36,37].

The validation studies of the FSFI scale showed its reliability and validity. Overall test-retest reliability coefficients were high for each of the individual domains (r = 0.79 to 0.86). A high degree of internal consistency was demonstrated by the Cronbach alpha value (0.963). The analysis of intraclass correlations showed a high degree of correlation between total and subscale results (r = 0.848–0.943, *p* < 0.001) [38].

The HADS questionnaire was used to determine the level of anxiety and depression. The questionnaire consists of two domains (anxiety and depression), each assessed in the range of 0 to 21 points. Women with a score of ≤7 did not show symptoms of depression or anxiety. A greater number of points indicated a higher level of depressive and anxiety behaviours, defined as mild (8–10 points), moderate (11–14 points), and severe (15–21 points) [18,39]. The validation studies of the HADS scale showed its reliability and validity. Spearman’s rank co-relation between the test items and the overall score of a given subscale was statistically significant (*p* < 0.01) and ranged from 0.41 to 0.76. The mean sensitivity and specificity were ≥0.80, similar to that of other self-assessment tools. Similar alpha coefficients were observed for the translated versions [40].

Strategies for coping with stress were assessed using the Mini-COPE questionnaire, which consists of 28 questions. They form 14 subgroups, including various strategies of coping with difficult situations. These include: active coping, planning, positive reframing and development, acceptance, sense of humour, turning to religion, seeking emotional support, seeking instrumental support, self-distraction, denial, venting, use of alcohol and psychoactive substances, discontinuation of actions, and self-blame. The results were analysed separately for each strategy, and their range was from 0 to 3 points. The higher the result, the more often a given strategy was adopted. The internal compliance of the Polish version of Mini-COPE was established based on a study of 200 patients aged 25–60 years. The half-time reliability was 0.86 (Guttman’s index 0.87). The consistency was satisfactory for most scales [41,42]. The respondents completed an original questionnaire, which included sociodemographic, health, and lifestyle questions.

The study was conducted in accordance with the standards of the Helsinki Declaration. It was approved by the Bioethics Committee of the Pomeranian Medical University (decision no KB-0012/47/04/2021/Z).

### 2.3. Statistical Analysis

The statistical analysis was performed with Statistica version 13.1. The study group was characterised taking into account the number of patients, their percentage share, mean, minimum and maximum, and standard deviation. The normality of the distribution was tested with the Shapiro–Wilk test. The relationships between the groups were analysed with the Student’s *t*-test and the Mann–Whitney U test. Correlation analysis was performed with the Spearman’s rho test. Nominal variables were tested with the chi-squared test. Results where *p* < 0.05 were considered statistically significant.

## 3. Results

### 3.1. Group Characteristics

A total of 860 patients were included in the study. The patients were characterised in terms of age, education, and the presence of habitual behaviours (smoking and drinking alcohol). The analysed factor was the course of the menstrual cycle, multiple pregnancies, and the occurrence of miscarriage. The mean (±SD) age in the study group was 33.1 ± 6.0 years, and in the control group was 32.6 ± 7.0 years. The characteristics of women with endometriosis compared to the healthy population are presented in Table 1.

The study group was characterised in terms of the clinical stage of the disease, the location of endometriosis lesions, the presence of accompanying symptoms, the method of diagnosis, and the areas of possible help, taking into account the age groups of 18–25, 25–35, 35–45, and over 45.

The greatest number of women (33.3%) suffered from stage 4 endometriosis. Slightly fewer (30.5%) persons were in stage 1 (Table 2).

In the 18–25 age group, the most common endometriosis foci were localised in the ovaries (67.39%), the peritoneum (36.96%), and the fallopian tubes (21.74%). The analysis of the results in the 25–35 age group was as follows: the endometriosis lesions were present in the ovaries (72.34%), fallopian tubes (31.91%), and peritoneum (30.93%). In the groups of 35–45 years and over 45 years of age, respectively, the data were as follows: ovaries (74% and 57.14%), peritoneum (37.33% and 14.29%) and fallopian tubes (31.33% and 14.29%). The most persistent complaint reported by the respondents in all age groups was pain during menstruation (group 18–25 years old: 80.43%, group 25–35 years old: 88.14%, group 35–45 years old: 84%, group over 45 years old: 57.14%) and in the pelvic region (age group 18–25: 73.91%, age group 25–35: 66.10%, age group 35–45: 63.09%, age group over 45: 57.14%). The method used to diagnose the presence of endometriosis in most cases was laparoscopic surgery (age group 18–25: 52.17%, age group 25–35: 59.32%, age group 35–45: 59.33%, age group over 45: 57.14%). One of the least used diagnostic methods was ultrasound examination (group 18–25 years old: 43.48%, group 25–35 years old: 34.32%, group 35–45 years old: 28%, group over 45 years old: 42.86%). Most respondents expressed their willingness to obtain assistance in the search for the optimal method of treatment (66.67%) and therapy financing (63.89%). The interest for aid and assistance in different areas of life varied across different age groups. The respondents aged 18–25 mainly expressed the will to receive help in treatment financing (67.39%) and choice of optimal treatment method (60.87%), in addition to psychological (50%) and dietary (50%) support. In the 25–35 age group, most women required information on the optimal method of treatment (66.53%), treatment financing (64.83%), and relapse prevention (56.36%). In the 35–45 age group, the greatest number of women wanted to receive help in the choice of optimal treatment method (66.67%), relapse prevention (62%), and treatment financing (59.33%). The data in the age group over 45 years old was slightly different. Help in relapse prevention and seeking specialist aid was expressed by 57.14% of women. The willingness to look for the optimal method was expressed by 42.86% of the respondents.

### 3.2. Quality of Life Assessment

The analysis of the results of individual questionnaires, both in the study group and the control group, is presented in Table 3. The levels of depressive and anxiety disorders, amounting to 8.78 ± 2.5 (group B) and 11.48 ± 3.0 (group C), respectively, were higher in the study group compared to the control group (*p* < 00.1). The mean concerning particular sexual functions in comparison with the study group was demonstrated to be higher in the control group. The exception was the domain of pain during intercourse. The analysis of the results of the Mini-COPE questionnaire revealed that women with endometriosis less frequently demonstrated the strategy of active coping (*p* = 0.012), and positive reframing and development (*p* < 0.001). They showed a lower sense of humour (*p* = 0.002), a greater need to seek instrumental support (*p* = 0.049), and discontinued their actions more often (*p* = 0.005).

In the Table 4, the correlations of the HADS and IPAQ questionnaires with other variables used in the study were assessed. In group B, there was a correlation between the occurrence of depressive disorders and sexual functioning of women in the overall score (*p* = 0.021), in addition to the specific domains (desire, excitement, satisfaction) and in the strategy to deal with stress (*p* = 0.003). In group C, there was a correlation between depressive disorders and sexual functioning in the overall score (*p* < 0.001), and in all domains, except for pain during intercourse, and the level of physical activity (*p* = 0.002). The level of anxiety was negatively correlated with the FSFI (*p* < 0.001). The same influence of anxiety on women’s sexual functioning was observed in group C (*p* < 0.001) as in group B. The observed changes concern both the total overall score of FSFI and all separate domains that were measured in the study. In the group of patients diagnosed with endometriosis, a relationship was found between the level of physical activity and the ability to cope with stress (*p* = 0.001).

Table 5 demonstrates the relationship between the level of physical activity and sexual functioning, depression and anxiety disorders, and strategies for coping with stress, taking into account the division according to endometriosis staging. A relationship was found between the level of physical activity and anxiety disorders (*p* = 0.015) and strategies for coping with stress, such as distraction (*p* = 0.007), and consumption of alcohol and other drugs (*p* = 0.029), among patients with the second stage of the disease. In patients with stage III endometriosis, a relationship between physical activity and active coping (*p* = 0.035), planning (*p* = 0.003), acceptance (*p* = 0.037), and distraction (*p* = 0.026) was found. Among women diagnosed with stage IV endometriosis, an association was observed between planning (*p* = 0.049), distraction (*p* = 0.008), and denial (*p* = 0.002).

After the regression modelling was performed, adjusted for age, education, and cigarette smoking (Table 6), a correlation between anxiety disorders (*p* = 0.014) and sexual functioning (*p* = 0.001), and the age of the patients was found. The level of education revealed a relationship between sexual functioning (*p* = 0.043) and the level of physical activity (*p* = 0.023).

## 4. Discussion

There are many studies in the available literature assessing the functioning of women diagnosed with endometriosis. This study is the first attempt at a multivariate analysis to assess the relationship between physical activity and individual variables, such as sexual function, anxiety, and depressive disorders, in addition to coping strategies, taking into account the stage of the disease.

Depression and anxiety are the most common disorders resulting from endometriosis [29,30,31,43,44,45]. In the present study, as in the study by Laganà et al. a higher level of depression and anxiety was found in women with endometriosis compared to healthy women [31]. Chen et al. confirmed the correlation between endometriosis and the presence of psychiatric disorders [30]. This study shows that there is no correlation between the symptoms of depression and anxiety and the stage of endometriosis. Other authors confirm this relationship [46,47,48]. A high level of psychopathological symptoms was more often diagnosed among women with pelvic pain [49,50,51,52,53]. Some authors have shown that pain may be the only cause of depression and anxiety among women with endometriosis [50,54]. However, the exact causality of this correlation could not be identified [46]. There are many confounding factors that may have influenced the obtained results. Age was an important aspect. Earlier studies have proven the influence of reduced oestrogen production in postmenopausal women on the relief of endometriosis symptoms [17]. Other variables are: duration of the disease, delay in diagnosis of endometriosis, social support, psychological aspects (e.g., stress, anxiety, depression, and family/work problems unrelated to the underlying disease), use of painkillers and opioids, infertility, pregnancy/successful delivery, response/non-response to treatment, patient’s place of residence, financial status, access to health care, use of complementary or alternative forms of treatment, and predisposition to pain/chronic pain syndromes [55].

Pain during and after intercourse is a common problem that reduces the quality of sexual life among women with endometriosis [56,57,58]. According to Vercellini et al. women with endometriosis more often experienced dyspareunia [59]. The present study showed differences in sexual functioning between women with endometriosis and women without endometriosis. Similar results were presented in the work by Fairbanks et al. [60]. According to Ferrero et al. localisation of the disease within the cruciate ligaments was associated with greater discomfort during intercourse [61,62]. Sexual problems significantly affect the quality of a relationship [63,64]. Hämmerli et al. proved that sexual problems were more common among couples in which the woman had endometriosis [65]. Fritzer et al. showed statistically significant correlations between sexual dysfunction and a greater sense of guilt towards the partner, in addition to a decreased sense of femininity [66]. This relationship may have been the basis for the intensity of depression and anxiety in women with endometriosis that was noticed in the present study. Similar observations were demonstrated by Youseflu et al. who found a relationship between the occurrence of anxiety disorders, depression, and the level of sexual functioning [67]. Graaff et al. showed that depression was a significant negative predictor of sexual functioning [68].

Women with endometriosis have a greater tendency to show panic symptoms and an increased perception of stress [69]. No other studies were found to assess stress-coping strategies in women with endometriosis, and this study is the first to demonstrate such a relationship. According to the results, women with endometriosis needed more instrumental support. They rarely showed a strategy of active coping and more often discontinued their actions.

The decline in physical activity among women with endometriosis may be associated with pelvic pain [70]. According to the authors, physical exercise is an important factor in reducing pain [71,72]. Zhao et al. proved that progressive muscle relaxation training is effective in alleviating pain, anxiety, and depression in women with endometriosis undergoing hormone therapy [73]. Stanton et al. demonstrated a positive effect of physical activity on sexual function [74]. No studies have been found to assess the impact of physical activity on coping strategies. In the present study, the relationship between physical activity and individual aspects of women’s quality of life was analysed for the first time, taking into account the clinical advancement of endometriosis.

### Limitations

Due to the ongoing Sars-CoV-2 coronavirus pandemic, direct access to patients was difficult, and they were eventually brought together via appropriate online forums. As a result of the respondents filling in online questionnaires themselves, there is a risk of an information error. It was not possible to check and correct, if needed, the answers to individual questions. Another difficulty in conducting the study was obtaining answers to questions considering the intimate aspects of women’s functioning.

## 5. Conclusions

The occurrence of endometriosis affects the functioning of women in multiple aspects of life. The sexual function of women with endometriosis may be affected by the presence of mental disorders. Physical activity may be a factor that modifies the strategies of coping with stress among women with particular clinical stages of endometriosis. 

## Figures and Tables

**Table 1:** Comparison of the demographic data of the test group and the control group.

Variable		Group B		Group C		*p*-Value
		M	Min–Max	M	Min–Max	
Age		33.1	19.0–55.0	32.6	18.0–50.0	0.235
		N	%	N	%	*p*-Value
Education	Primary	5	1.13%	3	0.71%	<0.001 *
	junior high school	2	0.45%	5	1.18%	
	vocational	4	0.91%	16	3.80%	
	secondary	106	24.14%	163	38.71%	
	Higher	322	73.34%	234	55.58%	
Menstrual cycle	stopped by medication	123	14.30%	28	3.26%	<0.001 *
	Regular	138	16.05%	172	20.00%	
	quite regular	119	13.84%	173	20.12%	
	irregular	53	6.16%	47	5.47%	
	I am bleeding all the time	6	0.70%	1	0.12%	
Occurrence of miscarriage	Yes	70	13.49%	65	12.52%	0.249
	No	177	34.10%	207	39.88%	
Multiple pregnancies	one child	172	43.11%	203	50.88%	0.746
	Twins	9	2.26%	14	3.51%	
	triplets or more	0	0.00%	1	0.25%	
Smoking cigarettes	Yes	62	14.12%	106	25.17%	<0.001 *
	No	377	85.87%	315	74.82%	
Drinking alcohol	does not drink	92	10.71%	71	8.27%	0.017 *
	occasionally	233	27.12%	210	24.45%	
	once a week	76	8.85%	75	8.73%	
	once a month	35	4.07%	58	6.75%	
	every day	2	0.23%	7	0.81%	

Group B—study group; Group C—control group; *p*-value—statistical significance; *—statistically significant value; N—number.

**Table 2:** The division of the study group according to endometriosis staging with regard to age groups.

Variable (Age)	Stage I	Stage II	Stage III	Stage IV	*p*-Value
18–25 years old	20	10	6	10	0.046 *
25–35 years old	71	42	50	73	
35–45 years old	40	23	25	62	
Above 45 years old	3	2	1	1	
Overall	134 (30.5%)	77 (17.5%)	82 (18.7%)	146 (33.3%)	

*p*-value—statistical significance; *—statistically significant value

**Table 3:** Intergroup analysis of the results of the questionnaires: Mini-Cope, FSFI, HADS, and IPAQ.

Variable		Group B				Group C				*p*-Value
		M	Min	Max	SD	M	Min	Max	SD	
**HADS**	Depression	8.78	2.00	18.00	2.50	5.08	0.00	18.00	3.15	<0.001 *
	Anxiety	11.48	5.00	18.00	3.00	8.66	0.00	20.00	3.90	<0.001 *
**FSFI**	Overall	21.30	0.00	46.50	7.04	23.89	1.20	34.40	6.97	<0.001 *
	Desire	1.88	0.00	4.80	1.30	2.78	0.00	4.80	1.28	<0.001 *
	Excitement	3.71	0.00	6.00	1.57	4.36	0.00	6.00	1.57	<0.001 *
	Lubrication	4.08	0.00	6.00	1.69	4.83	0.00	6.00	1.66	<0.001 *
	Orgasm	3.90	0.00	6.00	1.68	4.37	0.00	6.00	1.74	<0.001 *
	Satisfaction	3.43	0.00	5.60	1.40	3.80	0.00	5.20	1.27	<0.001 *
	Pain	3.51	0.00	6.00	1.56	2.21	0.00	6.00	1.30	<0.001 *
**IPAQ**		793.79	0.00	10,956.00	1123.05	834.02	0.00	14,680.00	1137.22	0.135
**Mini-COPE**	Active coping	1.89	0.00	3.00	0.83	2.05	0.00	3.00	0.74	0.012 *
	Planning	1.84	0.00	3.00	0.86	1.89	0.00	3.00	0.77	0.606
	Positive reframing and development	1.33	0.00	3.00	0.87	1.58	0.00	3.00	0.78	<0.001 *
	Acceptance	1.59	0.00	3.00	0.82	1.70	0.00	3.00	0.75	0.069
	Turning to religion	0.71	0.00	3.00	0.86	0.67	0.00	3.00	0.91	0.246
	Sense of humour	0.67	0.00	3.00	0.62	0.81	0.00	3.00	0.66	0.002 *
	Seeking emotional support	1.62	0.00	3.00	0.90	1.67	0.00	3.00	0.93	0.424
	Self-distraction	1.56	0.00	3.00	0.84	1.62	0.00	3.00	0.80	0.382
	Seeking instrumental support	1.55	0.00	3.00	0.88	1.43	0.00	3.00	1.03	0.049 *
	Denial	0.75	0.00	3.00	0.76	0.75	0.00	3.00	0.76	0.970
	Venting	1.42	0.00	3.00	0.71	1.49	0.00	3.00	0.73	0.216
	Use of alcohol and psychoactive substances	0.36	0.00	3.00	0.66	0.45	0.00	3.00	0.76	0.293
	Discontinuation of actions	0.79	0.00	3.00	0.71	0.67	0.00	3.00	0.70	0.005 *
	Self-blame	1.33	0.00	3.00	0.87	1.38	0.00	3.00	0.93	0.541

Group B—study group; Group C—control group; M—arithmetic mean; SD—standard deviation; Min—minimum; Max—maximum; *p*-value—statistical significance; *—statistically significant value

**Table 4:** Correlations between the results of the questionnaires.

Variable			Group B		Group C	
			R	*p*-Value	R	*p*-Value
**HADS**	Depression	FSFI-overall	−0.10971	0.021 *	−0.31206	<0.001 *
		FSFI-desire	−0.1109	0.020 *	−0.3004	<0.001 *
		FSFI–excitement	−0.1203	0.012 *	−0.2310	<0.001 *
		FSFI–lubrication	−0.0381	0.427	−0.2448	<0.001 *
		FSFI–orgasm	−0.0812	0.090	−0.2529	<0.001 *
		FSFI–satisfaction	−0.1051	0.028 *	−0.2860	<0.001 *
		FSFI-pain	0.0552	0.250	0.0839	0.086
		IPAQ	−0.01737	0.717	−0.14881	0.002 *
	Anxiety	FSFI-overall	−0.18758	<0.001 *	−0.33007	<0.001 *
		FSFI-desire	−0.1346	0.005 *	−0.2522	<0.001 *
		FSFI–excitement	−0.1203	0.012 *	−0.2806	<0.001 *
		FSFI–lubrication	−0.1586	0.001 *	−0.2408	<0.001 *
		FSFI–orgasm	−0.1451	0.002 *	−0.2915	<0.001 *
		FSFI–satisfaction	−0.2215	<0.001 *	−0.3041	<0.001 *
		FSFI-pain	0.2046	<0.001 *	0.2542	<0.001 *
		IPAQ	0.07447	0.119	−0.00489	0.920
**IPAQ**	FSFI-overall		−0.00926	0.847	0.02139	0.662
	FSFI-desire		0.0572	0.233	−0.0471	0.335
	FSFI–excitement		−0.0497	0.300	−0.0117	0.811
	FSFI–lubrication		−0.0085	0.860	−0.0061	0.901
	FSFI–orgasm		−0.0585	0.222	−0.0589	0.228
	FSFI–satisfaction		−0.0034	0.944	−0.0262	0.593
	FSFI-pain		0.0908	0.058	−0.0065	0.894
	HADS depression		−0.01737	0.717	−0.14881	0.002 *
	HADS anxiety		0.07447	0.119	−0.00489	0.920

Group B—study group; Group C—control group; *p*-value—statistical significance; R—correlation coefficient; *—statistically significant value

**Table 5:** Relationship between the level of physical activity and sexual functioning, ability to cope with stress, and the degree of anxiety and mood disorders, taking into account the clinical stage.

Variable		Stage I		Stage II			Stage III		Stage IV	
		R	*p*	R	*p*		R	*p*	R	*p*
**IPAQ**	**HADS**	Depression	0.00902	0.918	−0.02735	0.813	0.04737	0.672	−0.07507	0.368
		Anxiety	0.00211	0.981	0.27574	0.015 *	0.08742	0.435	0.03464	0.678
	**FSFI**		0.01889	0.828	0.07788	0.501	−0.06894	0.538	−0.04495	0.590
	**Mini-COPE**	Active coping	0.14862	0.087	0.03549	0.759	0.23342	0.035 *	0.09011	0.279
		Planning	0.06983	0.423	0.07719	0.505	0.32578	0.003 *	0.16344	0.049 *
		Positive reframing and development	0.00002	0.999	0.01418	0.903	0.19491	0.079	0.10674	0.199
		Acceptance	0.15535	0.073	0.06890	0.552	0.23033	0.037 *	0.01722	0.837
		Turning to religion	−0.00363	0.967	0.18934	0.099	−0.00890	0.937	0.12492	0.133
		Sense of humour	0.05783	0.507	0.10948	0.343	−0.05839	0.602	−0.00235	0.978
		Seeking emotional support	−0.04981	0.568	0.12528	0.278	0.08316	0.458	−0.01202	0.885
		Self-distraction	0.07032	0.419	0.30425	0.007 *	0.24565	0.026*	0.21765	0.008 *
		Seeking instrumental support	−0.01219	0.889	0.15562	0.179	0.14545	0.195	−0.05164	0.536
		Denial	−0.12039	0.166	0.02912	0.801	0.02451	0.827	0.25349	0.002 *
		Venting	−0.01942	0.824	−0.15412	0.181	−0.10949	0.327	0.05590	0.503
		Use of alcohol and psychoactive substances	−0.11449	0.188	0.24961	0.029 *	0.01804	0.872	−0.02620	0.754
		Discontinuation of actions	0.02184	0.802	0.09309	0.421	0.01248	0.911	0.12836	0.123
		Self-blame	−0.09049	0.298	0.08668	0.454	0.10695	0.339	0.07351	0.378

*p*-value—statistical significance; R—correlation coefficient; *—statistically significant value

**Table 6:** Multivariate logistic regression analysis.

Variable	Age				Education Level				Cigarette Smoking			
	β	*p*-Value	95% CI		β	*p*-Value	95% CI		β	*p*-Value	95% CI	
**HADS-depression**	0.047954	0.358	−0.05451	0.150421	−0.08742	0.094	−0.18985	0.015016	0.040992	0.439	−0.06304	0.145019
**HADS-anxiety**	−0.12881	0.014 *	−0.23178	−0.02583	−0.08084	0.123	−0.18378	0.022105	−0.05813	0.275	−0.16267	0.046417
**FSFI**	−0.14992	0.001 *	−0.24384	−0.05599	−0.09718	0.043 *	−0.19108	−0.00329	−0.02813	0.562	−0.12348	0.067228
**IPAQ**	0.074198	0.117	−0.01853	0.166922	−0.1074	0.023 *	−0.20009	−0.01471	0.063958	0.182	−0.03018	0.158093

Legend: *p*-value—statistical significance, Cl-confidence interval, β—standardized regression coefficient; *—statistically significant value.

## Data Availability

All data was collected in the Department of Medical Rehabilitation and Clinical Rehabilitation, Pomeranian Medical University, 71-210 Szczecin.
